# Health-Related Quality of Life and Service Barriers among Adults with Sickle Cell Disease in Saudi Arabia

**DOI:** 10.4314/ejhs.v33i5.13

**Published:** 2023-09

**Authors:** Nada Ahmed Al Sayigh, Marwa Mahmoud Shafey, Amal Ali Alghamdi, Ghada Fouad Alyousif, Fatma Amer Hamza, Zaenb Husain Alsalman

**Affiliations:** 1 Health Emergency Operation Center, Eastern Province, Saudi Arabia; 2 Department of Family and Community Medicine, College of Medicine, Imam Abdulrahman Bin Faisal University, Dammam, Saudi Arabia; 3 Department of Family and Community Medicine, College of Medicine, King Faisal University, Alahsa, Saudi Arabia

**Keywords:** Health-related quality of life, sickle cell disease, SF-36 item, adult, Saudi Arabia

## Abstract

**Background:**

The burden of sickle cell disease (SCD) is high in Saudi Arabia, with a significant impact on patients' quality of life (QoL). This study aimed to assess the health-related quality of life (HRQoL) among adults with SCD.

**Methods:**

A cross-sectional study was conducted among adults with SCD attending hematology clinics at Qatif Central Hospital in the Eastern Province of Saudi Arabia. The questionnaire included subsections to collect information from participants, including sociodemographic attributes, SCD characteristics, HRQoL based on SF-36, and opinions regarding barriers to service.

**Results:**

Among 272 SCD patients, the highest mean score of HRQoL was observed in the social functioning (SF) domain (65.0±23.4), whereas the lowest score was observed in the role limitations due to physical health (RP) domain (47.2±40.4). The mean score for participants' opinions regarding service provision was 19.27±4.68 (min-max:10–30), and only 24.6% had a positive opinion regarding the accessibility of service provision. A total of 38.6% of the respondents acknowledged shortcomings in the services offered by healthcare staff, and 43% identified weaknesses in communication with healthcare staff. Moreover, 40.1% agreed about feeling stigmatized about their condition. SCD patients who were <40 years old, males, had a university degree, had health insurance, waited <15 minutes before receiving health care, and had positive opinions regarding service provision were more likely to have better HRQoL scores.

**Conclusion:**

Adults with SCD exhibited low HRQoL in general, and different factors were related to low HRQoL scores. Counselling, empowerment, and improvement of doctor-patient communication are important strategies to improve healthcare provision, and consequently, HRQoL among adults with SCD.

## Introduction

Sickle cell disease (SCD) is a type of genetic blood disorder in which red blood cells have an abnormal hemoglobin structure that results in tissue hypoxia and organ failure (1). Patients with SCD exhibit signs and symptoms early in their childhood, which vary depending on the severity of the disease (2). The physical and psychological complications of SCD have an adverse impact on health-related quality of life (HRQoL) domains, which results in more daily demands, difficulties in fulfilling family and social activities, work, and educational requirements, as well as financial burdens (3). HRQoL is a multidimensional perception that reflects the effects of chronic disease and healthcare services on patients and their families (4,5).

Although SCD is associated with significant use of healthcare resources, patients with SCD frequently encounter barriers and challenges to accessing adequate care. SCD patients who have insurance will receive care in a timely manner; however, others may not be able to afford private services and may be compelled to attend overcrowded public hospitals (6). Moreover, the requirement for pain relievers on a regular basis might result in stigmatization (7). These healthcare service barriers for SCD patients vary depending on the patients' experiences and disease severity, which may include appointment registration, medication, transportation issues, or healthcare providers' attitudes, in addition to long waiting times and stigma (7,8).

Information about SCD prevalence in Saudi Arabia is still inconsistent and differs from region to region. According to statistical studies, the highest rate is found in the Eastern (Al-Ahsa and Qatif) and south-western (Al-Qunfudah and Jazan) regions of Saudi Arabia (9,10). Although SCD is an important public health problem in Saudi Arabia and HRQoL is considered an important health outcome measure, Saudi researchers have not given this topic the priority it needs (11). From both medical and health service perspectives, caring for people with SCD is complicated; training healthcare professionals, increasing awareness at an early age, and combating service provision barriers are essential strategies for improving HRQoL among SCD patients (7,12). Therefore, this study aimed to assess HRQoL and its determinants along with investigates the barriers to service provision among adults with SCD.

## Materials and Methods

Study design and target population: A cross-sectional study was conducted from March to October 2020 among adults with SCD aged 20 years or older who attended hematology clinics at Qatif Central Hospital in the Eastern Province of Saudi Arabia. This study was approved by the Imam Abdulrahman Bin Faisal University Institutional Review Board (IRB-PGS-2020-03-027).

**Sample size calculation:** The sample size was calculated at 384 by using the Epi Info statistical software (13). Assuming the proportion of adults' HRQoL is 50% at a confidence level of 95%, power of 80%, and a degree of precision of 5%.

Data sources/measurement: Data was collected using an Arabic self-administered questionnaire with four sections. The first section gathered sociodemographic information from the participants. The second section focused on SCD and health-related data, including the frequency of emergency department (ED) visits, frequency of admission, emergency room (ER) waiting times before the examination (categorized as <20 minutes or ≥20 minutes), and waiting times before receiving analgesia (categorized as <15 minutes or ≥15 minutes). The third section consisted of statements related to the accessibility of service provision. This included transportation, scheduling appointments, healthcare providers' attitudes and communication, ER waiting times before the examination and before receiving any analgesics, feelings of anxiety about privacy, overwhelming distress and urgency associated with the SCD condition, and experiences of stigmatization. The final section involved gathering data about HRQoL using a validated Arabic-translated RAND SF-36 form. Patients rated their HRQoL based on their feelings and perceptions across eight different components. The physical health measure included four scales: physical functioning (PF) (10 items), role-physical (RP) (4 items), bodily pain (BP) (2 items), and general health (GH) (5 items). The mental health measure included vitality (VT) (4 items), social functioning (SF) (2 items), role-emotional (RE) (3 items), and mental health (MH) (5 items) (14).

A pilot study was conducted to ensure the clarity of the questionnaire, and its reliability was assessed using Cronbach's alpha, which was estimated to be 0.82. Patients' privacy was assured, and the confidentiality of their information was maintained.

**Statistical analysis**: Data analysis was performed using IBM SPSS Statistics 24 (15). Categorical variables were reported as percentages and frequencies while mean, standard deviation, and median were used to report continuous variables. For assessing opinions regarding the accessibility of the service, 10 negative statements were scored on a scale from 1 to 3 (*totally agree, neutral*, and *disagree*). Raw scores (10–30), mean scores, and mean percent scores were calculated, and the mean percent scores were further categorized into negative, neutral, and positive opinions of service provision (< 50%, 50% to < 75%, and ≥ 75%, respectively).

For HRQoL, the 36-item SF scores were calculated and obtained according to the instructions and scoring rules for the RAND 36-Item Health Survey (SF-36). The number of questions directed to each health concept ranged from two (for SF and BP) to 10 (for PF), and the number of response options per question ranged from two (*no, yes*) to six (*none, very mild, mild, moderate, severe*, and *very severe*). Each question was given a score from 0 to 100, and each scale was scored from 0 (representing the least favorable health state) to 100 (representing the most favorable health state). A mean score was calculated for each domain, ranging from 0 to 100, with higher scores indicating a better outcome. A mean score of 50 was considered a normative value for all scales (16). Means and medians were compared between each variable and each HRQoL domain. The normal distribution of the sample was assessed with normality plots. The Mann-Whitney and Kruskal-Wallis tests were used to assess the differences between two or more groups of independent variables and the continuous dependent variable (HRQoL domains). Eight individual multiple linear regression models were conducted to analyze the relationship between candidate independent variables and the prediction of HRQoL. The results of the multiple regression analysis were summarized using β coefficients and *p*-values. The level of significance was set at a *p*-value of < 0.05.

## Results

**Participants' characteristics**: The total number of participants was 272 (a response rate of 71%). The highest percentage (44.1%) was among participants aged 20 to less than 30 years. Females outnumbered males (56.3% vs. 43.8%), and 53.7% of the participants were married. A significant proportion of the participants had attained a bachelor's degree (55.9%), while 37.5% held secondary certificates. The vast majority of the participants were of Saudi nationality (99.6%) and lived in Al Qatif (97.8%). Approximately, half of them were unemployed (52.2%), and more than two-thirds did not have health insurance coverage (69.1%). Slightly more than half (54.8%) had an income of < 5000 SR while only 13.6% had an income of > 10000 SR.

**Figure 1:**
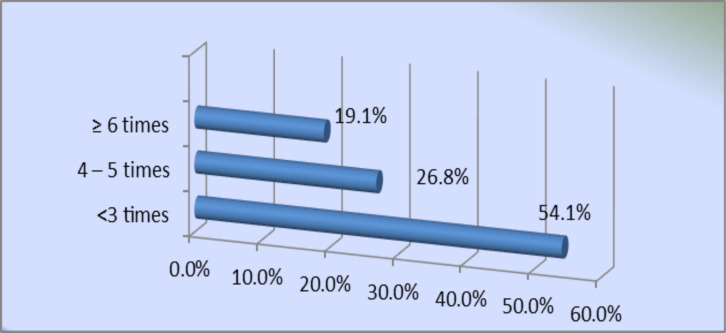
Distribution of Participants according to the number of Painful episodes in the last 6 months

**SCD and health-related characteristics**: Table 1 shows that 64.7% of the adults with SCD reported one to three ED visits within the last 6 months while those, who reported ≥ 6 ED visits, were only 8.8%. Those who reported ≥ 20 minutes of ER waiting time before the examination were 58.5%, and 68.4% reported ≥ 15 minutes of waiting time before receiving analgesics. Participants who reported no admissions in the last 6 months were 44.9%, and only 3.3% required ≥ 5 admissions. Less than half of the participants (42.3%) required only one hematology clinic visit. In Fig. 1, it can be observed that approximately 19.1% of adults with SCD reported experiencing ≥ 6 painful episodes in the previous 6 months while 54.1% reported having ≤ 3 painful episodes.

**Table 1:** Distribution of adults with SCD according to disease characteristics

Variable	Frequency (n = 272)	Percentage (100 %)
**Number of ED visits in the last 6 months**		
1-3 times	176	64.7%
4– 6 times	72	26.5%
>6 times	24	8.8%
**ER waiting time before examination**		
< 20 minutes	113	41.5 %
≥ 20 minutes	159	58.5 %
**Waiting time before receiving analgesics**		
< 15 minutes	86	31.6%
≥ 15 minutes	186	68.4 %
**Number of admissions in the last 6 months**		
None	122	44.9 %
1-2 times	82	30.1 %
3 – 4 times	59	21.7%
≥ 5 times	9	3.3 %
**Number of visits to haematology clinic in the last 6 months**		
None	74	27.2 %
Once	115	42.3 %
2 – 4 times	75	27.6 %
≥ 5 times	8	2.9 %

**Patients' opinions regarding barriers to service provision**: Nearly two-thirds of the participants agreed about the long waiting times in the ER before the examination (64.7%). Similarly, 65.1% of the participants agreed about the long waiting times before receiving any analgesics. Those who agreed about weaknesses in services provided by healthcare staff constituted 38.6%, and less than half (43%) agreed about weaknesses in communication with healthcare staff. Around one-third agreed about difficulties in dispensing medications and registering appointments (34.2% and 32.8%, respectively), whereas only 18% agreed about experiencing transportation difficulties. Regarding the feelings of adults with SCD, 40.1% of them agreed about feeling stigmatized, 25.7% agreed about feeling urgency and distress regarding their medical state, and 23.2% agreed about feeling anxious regarding their health privacy (Table 2).

**Table 2:** Distribution of Participants According to their Opinion towards Accessibility of Service-Provision

Barrier to service-provision	Agree		Neutral		Disagree	

	No.	(%)	No.	%	No.	%
**There is difficulty in transportation when needing hospital**	49	(18 %)	91	(33.5%)	132	(48.5%)
**There is difficulty in registering appointments**	89	(32.8%)	82	(30.1%)	101	(37.1%)
**There is difficulty in dispensing medication**	93	(34.2%)	83	(30.5%)	96	(35.3%)
**Waiting time in ER before examination is long**	176	(64.7%)	71	(26.1%)	25	(9.2%)
**Waiting time before receiving any analgesics is long**	177	(65.1%)	70	(25.7%)	25	(9.2%)
**There is weakness in services provided by healthcare staff**	105	(38.6%)	95	(34.9%)	72	(26.5%)
**There is weakness in communications with healthcare staff**	117	(43%)	85	(31.3%)	70	(25.7%)
**I feel anxious about patient's privacy**	63	(23.2%)	83	(30.5%)	126	(46.3%)
**I feel disparate about my health condition**	70	(25.7%)	79	(29.4%)	122	(44.9%)
**I feel stigmatized by the community due to my SCD condition**	109	(40.1%)	84	(30.9%)	79	(29.0%)

The mean score for participants' opinions regarding service provision was 19.27 ± 4.68 (min-max: 10–30); the mean percent score was 64.24 ± 15.62. According to the mean percent score categorization, only 24.6% had a positive opinion regarding the accessibility of service provision while the majority (58.1%) had a neutral opinion and 17.3% had a negative one.

**Figure 2:**
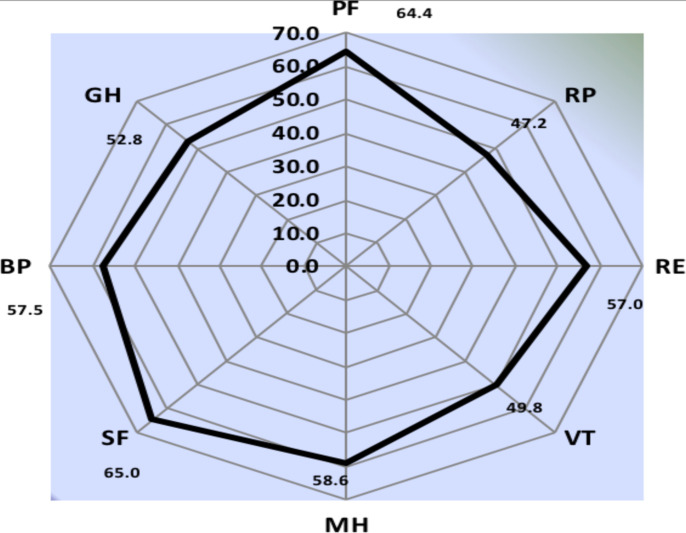
Spydergram for Different Domains of HRQoL-36 among Adults with SCD Abbreviations: Physical Functioning (PF), Role Limitations due to physical health (RP), Role limitation due to emotional problems (RE), Energy/Fatigue(VT), Mental Health (MH), Social Functioning (SF) Pain (BP) and General Health (GH)

**SCD Patients' HRQoL**: Figure 2 shows the mean score values of different domains of HRQoL among SCD patients. The highest score was observed in the SF (65.0 ± 23.4) and PF (64.4 ± 24.5) domains. The lower score was observed in the RP domain (47.2 ± 40.3)

**Bivariate analyses**: Table 3 shows that there was a statistically significant difference in age across HRQoL domains except for SF and GH. Male participants had higher scores in several HRQoL domains (PF, MH, SF, BP, and GH). Differences were statistically significant in PF and BP (*p* < 0.05). Employed individuals and those with medical insurance reported higher mean scores across all domains of HRQoL, although the differences were not statistically significant. Adults with higher incomes reported higher HRQoL mean scores in MH, SF, and BP. No differences in HRQoL mean scores have been reported in relation to ER waiting times before examination. Meanwhile, HRQoL mean scores in all domains were higher among adults who reported waiting times of < 15 minutes before receiving analgesia, and statistically significant differences were found in the VT and SF domains. Among adults with SCD, those who reported no hospital admissions had significantly higher HRQoL domain mean scores, except for PF. Additionally, higher HRQoL domain mean scores were reported among adults with fewer ED visits. As the number of painful episodes increased, the HRQoL domain mean scores decreased. Adults with SCD who had a positive opinion of service accessibility had significantly higher HRQoL scores than those with a negative opinion.

**Table 3:** Differences in HRQoL Domains Mean scores according to Sociodemographic, SCD Characteristics, and Opinion among Adults with SCD

Independent Variables	SF-36 domains							

	PF	RP	RE	VT	MH	SF	BP	GH
**Age (years)**								
20-30	69.29	53.75	58.88	52.79	59.30	66.35	61.75	54.62
30 - <40	58.10	38.94	48.42	44.26	53.97	61.97	51.81	49.89
40 - <50	63.61	43.61	69.50	52.02	65.10	67.02	55.85	52.44
≥50	68.50	62.50	56.66	56.50	64.40	68.75	67.00	59.00
K-W p value	0.004*	0.042*	0.034*	0.006*	0.004*	0.610	0.037*	0.094
**Gender**								
Male	69.41	43.06	55.74	47.60	58.84	65.44	60.22	53.43
Female	60.45	50.32	57.95	51.53	58.35	64.49	53.88	51.89
M-WU p value	0.001*	0.169	0.565	0.125	0.921	0.861	0.044*	0.383
**Income (SR)**								
<5000	63.28	42.95	53.46	47.85	55.91	61.32	52.91	51.07
5000–10000	67.96	54.65	61.62	53.19	62.41	71.22	63.69	55.58
>10000	60.40	46.62	60.36	49.86	60.75	65.54	61.21	52.97
K-W p value	0.473	0.182	0.304	0.136	0.035*	0.019*	0.008*	0.154
**Waiting time before analgesics**								
< 15 minutes	67.03	53.77	59.68	53.54	60.55	69.76	61.01	54.24
≥ 15 minutes	63.14	44.08	55.73	48.09	57.74	62.83	55.80	52.06
M-WU p value	0.341	0.067	0.473	0.038*	0.490	0.020*	0.109	0.373
**Number of Admissions**								
None	70.13	56.96	66.39	55.20	64.36	73.46	68.83	57.04
1-2	63.60	45.73	56.50	47.74	56.53	62.04	54.08	51.70
3-4	62.06	32.62	41.24	42.88	50.64	53.60	40.46	46.44
≥ 5 times	43.75	22.22	37.03	41.11	52.44	52.77	45.27	45.55
K-W p value	0.350	<0.001*	<0.001*	<0.001*	<0.001*	<0.001*	<0.001*	<0.001*
**Number of ED Visits in last 6 months**								
1 -3	67.41	53.97	62.12	54.20	62.75	69.88	65.61	55.99
4-6	59.79	35.76	49.07	42.36	50.72	57.98	46.42	48.26
>6	55.83	31.25	43.05	40.00	52.16	50.52	30.72	42.50
K-W p value	0.001*	0.001*	0.010*	<0.001*	<0.001*	<0.001*	<0.001*	<0.001*
**Pain Episodes in last 6 months (episodes)**								
< 3	70.95	58.16	71.42	57.61	64.43	72.95	69.52	58.70
3 - 6	57.19	34.24	41.09	38.90	51.34	58.04	46.917	46.57
> 6	55.86	34.31	38.46	43.07	52.46	52.40	38.12	44.61
K-W p value	<0.001*	<0.001*	<0.001*	<0.001*	<0.001*	<0.001*	<0.001*	<0.001*
**Opinion towards accessibility of service-provision**								
Negative	51.49	30.32	31.21	39.68	46.63	49.46	46.06	42.55
Neutral	67.82	39.63	41.32	51.45	59.72	67.48	59.06	54.52
Positive	65.29	39.22	73.63	53.05	64.47	70.14	57.50	55.74
K-W p value	0.003	0.002	<0.001	0.001	<0.001	<0.001	0.007	<0.001

* p-value significant <0.05

Abbreviations: Physical Functioning (PF), Role Limitations due to physical health (RP), Role limitation due to emotional problems (RE), Energy/Fatigue(VT), Mental Health (MH), Social Functioning (SF) Pain (BP) and General Health (GH).

**Regression analysis**: Table 4 presents the results of the multiple linear regression analysis. The increasing age intervals of < 40 years were associated with lower mean scores in the MH domain (β coefficient: 7.571, *p*=0.006). Male gender was associated with higher mean scores in the PF domain (β coefficient: 10.783, *p*<0.001), and mean scores of RE and MH were likely to increase among adults holding a university degree (*p*<0.05). Having health insurance was associated with higher mean scores in the PF and GH domains (*p*<0.05). Mean scores in the VT domain tended to increase with a waiting time of <15 minutes (β coefficient: 6.499, *p*= 0.019). Higher mean scores in the PF, MH, SF, and BP domains were likely to occur with no admission, whereas higher HRQoL mean scores in all domains were likely to occur with <3 painful episodes (*p*<0.001). Positive opinions toward service provision were likely to result in higher mean scores in the RE and MH domains (*p* <0.05).

**Table 4:** Linear Regression Analysis Models of Mean Scores of HRQOL SF36 Domains and Sociodemographic, SCD Characteristics, among adults with SCD

Independent variables	SF-36 domains, Standardized β coefficients, and p value							

	PF	RP	RE	VT	MH	SF	BP	GH
**Age (Ref: ≥40 years)**	0.708	1.931	-11.767	-5.222	-7.571	-2.154	2.845	-0.709
<40 years	0.853	0.675	0.067	0.063	0.006*	0.542	0.458	0.779
**Gender (Ref: Female)**	10.783	-5.688	3.253	-2.846	1.061	-0.056	-3.593	0.685
Male	<0.001*	0.280	0.533	0.230	0.644	0.984	0.250	0.739
**Education(Ref:<University)**	2.805	3.060	10.368	4.295	5.440	4.005	1.132	2.964
University	0.355	0.551	0.043*	0.064	0.016*	.155	0.710	0.140
**Job (Ref:Un-employed)**	.901	6.732	-5.904	0.139	-0.038	5.750	2.758	-2.493
Employed	0.771	0.201	0.258	0.953	0.987	0.046*	0.377	0.225
**Income (SR) (Ref:<10000)**	-6.133	-6.786	-6.342	-5.512	-4.357	-6.634	0.037	-0.855
≥10000	0.173	0.373	0.402	0.109	0.191	0.112	0.946	0.774
**Health Insurance (Ref:Non-insured)**	6.955	4.637	7.861	0.890	0.388	1.712	1.644	5.840
Insured	0.025*	0.378	0.132	0.707	0.866	0.551	0.598	0.005*
**ER waiting time before examination (Ref≥20min)**	-2.137	-9.444	-6.181	-1.739	-2.750	5.756	-0.396	-2.281
< 20 minutes	0.514	0.090	0.262	0.487	0.257	0.059	0.904	0.293
**Waiting time before receiving analgesics (Ref: ≥15min)**	5.435	12.020	3.395	6.499	3.919	3.442	3.956	1.709
< 15 minutes	0.133	0.050	0.579	0.019*	0.144	0.304	0.276	0.474
**Number of Admissions in the last 6 months (Ref: ≥1)**	7.667	8.313	2.759	1.933	4.777	7.920	10.004	2.106
None	0.015*	0.117	0.600	0.417	0.040*	0.007*	0.002*	0.309
**Number of ER Visits in last 6 months (Ref: >4)**	2.631	-0.234	-4.697	-1.864	-2.416	4.092	11.653	3.418
1 -3	0.621	0.979	0.600	0.647	0.586	0.407	0.030*	0.372
**Pain Episodes in last 6 months (episodes) (Ref: >4)**	10.947	19.683	5.437	15.552	9.308	13.409	19.941	11.158
< 3	<0.001*	<0.001*	<0.001*	<0.001*	<0.001*	<0.001*	<0.001*	<0.001*
**Opinion level Regarding access of service-provision**								
**(Ref: Neutral/Negative)**	3.606	8.214	5.813	2.766	7.694	5.471	4.100	4.462
Positive	0.298	0.162	<0.001*	0.295	0.003*	0.089	0.239	0.052

* p-value significant <0.05

Abbreviations: Physical Functioning (PF), Role Limitations due to physical health (RP), Role limitation due to emotional problems (RE), Energy/Fatigue(VT), Mental Health (MH), Social Functioning (SF) Pain (BP) and General Health (GH).

## Discussion

In Saudi Arabia, SCD is a highly prevalent disease with numerous complications that impair patients' HRQoL (12). This study aimed to assess HRQoL among adults with SCD and identify barriers to health services.

**HRQoL and related factors**: Adults with SCD in the current study experienced poor HRQoL scores in all eight domains; these results align with the findings from prior SCD studies conducted in Saudi Arabia and other settings (12,13,17,18). Despite the poor quality of life (QoL) among patients with SCD, little research has been done to identify the determining factors.

According to our study age was significantly associated with all HRQoL domains except SF and GH, and age < 40 was a significant negative predictor of MH. This significant association between age and HRQoL was reported in other Saudi research studies involving SCD and thalassemia patients (19,20). Additionally, males scored higher in the PF, MH, SF, BP, and GH domains, and gender had a statistically significant association with PF and BP. It is noteworthy that our findings contradict previous local studies among adults with SCD, as no relationship between gender and HRQoL scores was identified (11,12).

Education, employment, insurance, and income are important socioeconomic determinants of health and HRQoL. Adults with higher levels of education are less likely to experience financial hardship, have better job prospects and social standing, and have greater access to resources, all of which contribute to improved health. Educated people tend to value their mental and physical health, which leads to better QoL. Although the Saudi population receives free healthcare services, having insurance can provide patients with a sense of security and offer them access to a wider range of facilities. In our study, the HRQoL score of adults with SCD who had a university degree was higher, and a university degree was a significant predictor of RE and MH. Ahmed et al. (11) reported that patients with better scores on the RP, RE, and VT were university graduates. Furthermore, in our study, insured individuals or those who were employed had higher HRQoL scores in most domains (except GH), and insurance was a significant predictor of PF and GH. Khaled et al. (12) also found that insurance did not significantly impact physical HRQoL outcomes, but employed adults with SCD reported better scores in RP, VT, and MH than unemployed patients. Similarly, Ahmed et al. (11) reported that employed patients tended to have better scores in the VT and pain domains than unemployed patients. Moreover, our study found that higher income was associated with better HRQoL (MH, SF, and BP), which is consistent with a local study conducted among adolescents with SCD (20). However, income was not included as an independent predictor in the regression model, with employment being the sole independent predictor of SF.

The current study found that patients who waited < 15 minutes to receive analgesia had higher HRQoL scores, which is supported by the fact that 65.1% of the participants agreed that waiting times are long and perceived them as a barrier to service provision, ultimately affecting their HRQoL. The delay in obtaining analgesia may be attributed to the complex nature of SCD pain, which is subjective and challenging to assess objectively. Current HRQoL scores were higher with no admissions and lesser with ≥ 5 admissions in the last 6 months. The regression model clearly indicated that no admissions significantly predicted PF, MH, SF, and BP. Moreover, all HRQoL domain scores were significantly higher among individuals with fewer ED visits (1-3 times) within the previous 6 months, which is consistent with other findings (5, 21).

It is apparent in the present study that as the number of painful episodes increased, the HRQoL domain scores decreased. This finding is consistent with a study by Aljaouni (17). Pain is the most common complication of SCD, and according to our study, nearly one-fifth (19.1%) of our SCD patients had more than six episodes of pain in the previous 6 months, which was also reported in different studies (21,22,23).

**Barriers to service provision**: Regarding healthcare service barriers, adults with SCD experience challenges in managing the frequent episodes of SCD pain, which often leads to their reluctance to seek healthcare due to the stigma associated with their condition (24). In our study, nearly half of the adults with SCD agreed that they felt stigmatized by the community due to unexpected episodes of pain. Additionally, 43% agreed that there is a weakness in communication with healthcare staff, and nearly a third agreed about the inadequacy of services provided by healthcare staff. A study by Kanter et al. (25) demonstrated that respondents expressed higher satisfaction with their usual care physician compared to their experiences in the ED, and there was a correlation between experiencing severe pain or a lack of empathy from clinicians and negative perceptions of the quality of care.

Patients with a negative opinion regarding access to service provisions had significantly lower HRQoL scores in all domains. Therefore, it is crucial to empower and provide counseling to patients, as it can play a significant role in shifting their attitudes toward their disease and improving their perceptions of HRQoL.

Despite the efforts made by the government to combat this inherited disease through initiatives such as the premarital screening program and free genetic counseling, which have effectively reduced its incidence, there is still a need for additional programs to enhance healthcare access (26). Extensive training for healthcare providers concerning SCD and the implementation of broad social programs to raise awareness within the community will help overcome SCD-related service barriers and promote better healthcare outcomes.

Although this study is the first to be conducted in the region, it has some limitations. The data collection period was impacted by COVID-19 restrictions, which resulted in the conversion of clinics to virtual settings. This may have affected the response rate, and it should be noted that the study only included participants from hematology clinics, which may not fully represent the entire population of adults with SCD. Furthermore, QoL is a subjective issue that is perceived differently by each individual, which could result in self-reported bias. The cross-sectional design did not allow for the inference of causal effects, and the use of HRQoL scales may have limitations. Therefore, a more robust design, such as case-control studies, is recommended for future research on QoL.

In conclusion, adults with SCD generally exhibited low levels of HRQoL. Different sociodemographic, SCD, and health factors were found to be associated with low HRQoL scores. Saudi adults with SCD in this study reported barriers regarding accessibility to service provision. These results suggest that interventions aiming at improving PF, VT, BP, and MH might contribute to attaining high levels of QoL in adults with SCD. Counseling, support groups, empowerment, self-help programs, improved doctor-patient communication, and advocacy efforts are important strategies to improve the accessibility of healthcare provision and, consequently, HRQoL.
